# Gynaecological Screening for Cervical and Vulvar Malignancies in a Cohort of Systemic Sclerosis Patients: Our Experience and Review of the Literature

**DOI:** 10.1155/2015/761867

**Published:** 2015-10-18

**Authors:** M. Colaci, D. Giuggioli, G. Cassone, C. Vacchi, F. Campomori, F. Boselli, M. Sebastiani, A. Manfredi, C. Ferri

**Affiliations:** ^1^Chair and Rheumatology Unit, Medical School, Azienda Ospedaliero-Universitaria, Policlinico di Modena, University of Modena and Reggio Emilia, Via del Pozzo 71, 41100 Modena, Italy; ^2^Institute of Obstetrics and Gynecology, Oncology Prevention Unit, University of Modena and Reggio Emilia, Modena, Italy

## Abstract

*Background*. Increased incidence of cancer was frequently reported in scleroderma (SSc), but no association with gynaecological malignancies was described in literature. *Objectives*. To investigate gynaecological neoplasms in SSc patients. *Methods*. In this cross-sectional analysis, we evaluated 80 SSc patients, living in the same geographical area. We considered all patients undergoing gynaecological evaluation, including pap test as screening for cervical cancer, between January 2008 and December 2014. *Results*. 55 (68.7%) patients were negative and 20 (25%) presented inflammatory alterations, while cancer or precancerous lesions were found in 5 (6.2%) cases (2 showed cervical cancer (one of them in situ), 1 vulvar melanoma, 1 vulvar intraepithelial neoplasia, and 1 endocervical polyp with immature squamous metaplasia). The frequency of cervical cancer in our series seems higher in comparison to the incidence registered in the same geographical area. The presence of atypical cytological findings correlated with anti-Scl70 autoantibodies (*p* = 0.022); moreover, the patients with these alterations tended to be older (median 65, range 46–67), if compared to the whole series (*p* = 0.052). *Conclusions*. A relatively high frequency of gynaecological malignancies was found in our SSc series. In general, gynaecological evaluation for SSc women needs to be included in the routine patients' surveillance.

## 1. Introduction

Increased incidence of malignancies was widely reported in systemic sclerosis (SSc) [1]. In particular, a strong association with lung cancer and, less frequently, with haematological neoplasms was described [2]. As for other autoimmune diseases, it is not surprising that the chronic inflammation and the abnormal tissue reparative processes increase the risk of malignancy, particularly in target organs such as sclerodermic lungs. Nonetheless, a relation with breast cancer was suggested, even if it remains controversial, since only a few epidemiological studies found it [3]. By contrast, an association with the cancer of the uterine cervix was not reported in literature, considering that cervical uterus malignancy is one of the most frequent cancers in women worldwide [4]. Because of its high frequency, public health programs of screening for cervical cancer have been established in several countries, in order to identify precancer lesions or to treat the disease at an earlier stage [5]; the conventional cytological Papanicolaou test (pap test) is recommended every 3 years for all women between 21 and 65 years old [6].

The aim of the present study is to investigate the results of pap test in a cohort of SSc women, who underwent the screening for cervical cancer.

## 2. Patients and Methods

In this cross-sectional analysis, we included all SSc patients who underwent gynaecological screening for malignancies, including pap test, during the period between January 1, 2008, and December 31, 2014. Namely, we evaluated 80 women (mean age 51.2 ± 12 years and disease duration 7.9 ± 5.8 years at the end of the study), referring to our University-Based Rheumatology Centre, living in the same geographical area of the province of Modena (Northern Italy). In all cases the diagnosis of SSc was made by trained rheumatologists; anyway, all patients fulfilled the 2013 ACR/EULAR classification criteria for SSc [7].

Clinical records of epidemiological, clinical, serological, and instrumental data of all SSc patients along with analytic reports of the gynaecological evaluation were available for the study. Epidemiological data on cervical cancer were available online from Tumour Registry of the Province of Modena [8].

Statistical analysis was performed in order to investigate the possible associations between SSc parameters and cytological findings at pap test. Values are given as mean ± SD for normally distributed variables, or as median (range) for not normally distributed variables. Group values and proportions were compared by univariate analysis of variance (ANOVA) and Fisher's exact test, respectively. The significant level adopted was <5%.

## 3. Results

Eighty patients presented at least one cytological evaluation of the cervix at the end of the study period. At pap test evaluations, 55 (68.7%) patients were negative and 20 (25%) presented inflammatory alterations consistent with chronic cervicitis, while atypical cells related to cervical cancer were found in 2 (2.5%) cases, namely, a carcinoma* in situ* (CIN III) in one and invasive malignancy in the other one. In the remaining 3 patients the histological evaluation revealed a vulvar melanoma, a vulvar intraepithelial neoplasia (VIN III), and an endocervical polyp with immature squamous metaplasia, respectively. Overall, 5/80 (6.5%) SSc patients showed precancerous/cancerous lesions of the cervix or external genital organs (Table 1).

The age of our SSc patients with neoplastic lesions was 65 years (range: 46–67). Data from the Cancer Registry of our province [8] show that the higher incidence of cervical cancer regards the age groups 30–44 and 45–59. Therefore, our SSc patients with cancer were older than what we expect based on our reference population.

The frequency of cervical cancer in our series (2 cases) seems to be higher than expected, considering the incidence registered in the same geographical area and in the same time period (standardized rate 8.0 cases, 95% CI 5.2–10.7, out of 100,000 inhabitant-years) [8]. In fact, in the province of Modena, the cumulative risk to develop a cervical cancer during the entire life (0–74 years) is 0.6%, with a median age at diagnosis of 51 years [8]; thus, in our series of 80 patients followed up for 7.9 ± 5.8 years, we should expect less than 1 case (0.48 cases) of malignancy of the cervix. However, both the limited SSc series evaluated and the low number of cervical cancers observed did not permit obtaining a standardized incidence ratio (observed/expected events, SIR) with statistical significance.

In addition, the presence of atypical cytological findings correlated with serum anti-Scl70 autoantibodies (4/5 versus 19/75; *p* = 0.022), while patients with these alterations tended to be older (median 65, range 46–67), if compared to the whole series (*p* = 0.052). No statistical associations with skin or visceral SSc manifestations, smoking history, or treatment with immunosuppressors were found.

## 4. Review of the Literature

To the best of our knowledge, no other studies in literature focused on cervical or vulvar cancer in SSc. Nonetheless, sporadic cases of these types of tumours are frequently cited in the list of the malignancies found during registry-based or cohort studies (Table 2) [9–18].

In a study regarding a large SSc series [9] seen at the Mayo Clinic between 1959 and 1975, 10 cases of cervical cancer were reported; curiously, in 5 cases the diagnosis of the tumour and the diagnosis of SSc were made in the same year, suggesting possible paraneoplastic cases. One case of vulvar carcinoma was also reported.

In a population-based cohort of 441 SSc patients from South Australia [12], 5 cervical cancers were signalled out of 90 cases of neoplasms registered. However, authors did not evaluate this finding by statistical analysis because of the small number of patients developing this malignancy. In another registry-based study on 538 SSc patients from the Detroit metropolitan area [13], 3 women with cervical cancer were found, 1 invasive and 2* in situ*, in the absence of significantly increased incidence compared to general population. In a study evaluating a large series of 769 SSc patients prospectively followed up between 1987 and 2002 for the development of cancer [14], cervical cancer was recorded in 4/62 cases of malignancy observed after SSc diagnosis; one tumour of the vulva was also noticed. Again, the incidence of cervical-vulvar cancer observed in this patients series (5/769, 0.6%) was quite comparable to that reported in general population. Consistently, in the Danish nationwide population-based cohort study by Olesen et al. [15], among 162 cases of cancer in 1,586 female SSc patients, 9 cases of cervical cancer were observed (SIR 1.5, 95% CI 0.7–2.8). No cases of cervical cancer were registered in 2 large cohort studies in Japan [21] and Taiwan [22].

On the contrary, data of 218 SSc patients followed up in a Hungarian University-Based Centre [18] evidenced 10 patients with malignancies; one of them was a 53-year-old woman with cervical cancer. Authors indicated that the SIR versus general population was 37.0, but the interval of confidence was not calculated, so that the finding cannot be reliable.

Two recent studies [19, 20] on large SSc cohorts from the databases of highly specialised centres identified few cases of gynaecological cancers; however, these studies were designed for other purposes than the analysis of the incidence of the malignancies.

In 2009, Bernatsky et al. [23] investigated the history of an abnormal pap test by self-administrated questionnaire in a Canadian multicentre cohort of 320 SSc women. The lifetime prevalence of an abnormal pap test was 25.4%, compared to 13.8% reported in the Canadian general population; moreover, a significant positive association with diffuse skin subset, smoking history, and younger age was noted. Even if a detection bias could influence these findings (SSc patients may be more likely to be aware of comorbidities than general population), authors concluded that it seems prudent to ensure adequate attention to cervical cancer screening for SSc women.

## 5. Discussion

We evidenced an apparently high frequency of cancerous lesions of the cervix or the vulva in our cohort of SSc patients. The increase of the incidence of cervical/vulvar cancer in SSc is not reported by the world literature; however, the present study is the first to specifically investigate the incidence of gynaecological (cervical and vulvar) tumours in patients with SSc, while the other studies found in literature (Table 2) are focused on research of every type of tumours (mainly infiltrating and life-threatening cancers); nonetheless, they are characterized by heterogeneous designs and are based on different population subsets. Therefore, it could be assumed that in SSc women the appearance of cervical or vulvar cancers, particularly* in situ* lesions (i.e., CIN and VIN III), could be underestimated in large cohort or registry studies. In fact, in our series, 3/5 SSc patients with abnormal cytological tests did not present cervical cancer, but other pathological conditions that were generally excluded from the studies cited above, because of their low frequency.

Given these considerations, even if the cervical cancer, as specific clinical entity, may be considered as frequent as in the general population, periodic gynaecological screening for (pre)cancerous lesions of female genital organs should be carried out, since their occurrence might be not so uncommon in the course of SSc.

Pap smear abnormalities were also searched in other autoimmune disorders: in a case-control study [24] on 118 women affected mainly by systemic lupus erythematosus and rheumatoid arthritis, the frequency of abnormal pap tests was significantly higher in the patients (8.1% and 9.3%, resp.) compared to controls (1.7%); authors concluded that it might be an association between the autoimmune diseases and occurrence of (pre)cancer lesions of the cervix.

The main known trigger of cervical cancer is the human papillomavirus (HPV); in particular, a few specific virus genotypes are considered carcinogenetic [25]. Consequently, an increased risk of cervical cancer in SSc women, if confirmed, might indicate an increased infection/persistence of HPV in scleroderma patients. In this regard, no consistent data may be found in literature. Indeed, only one study [26] investigated the presence HPV infection and/or dysplasia in a small cohort of 31 SSc women; comparable frequencies of the virus versus healthy controls were noticed, but infection by 2 or more HPV types was 2 times more frequent in SSc patients. The possibility of a link between SSc and HPV needs to be deeply studied.

Finally, the possibility of a selection bias with the consequent risk to overestimate cervical cancer incidence cannot be excluded. However, our data on pap test regarded less than 2-thirds of the SSc women population from province of Modena. Yet, considering the findings of the Italian National Centre of Epidemiology, Surveillance and Health Promotion in the “Passi 2012” study [27], more than 75% of the women interviewed affirmed that they underwent a pap test in the last 3 years. This discrepancy points out to the possible risk that SSc patients, who are affected by a chronic disease with necessity to undergo a number of clinical and instrumental exams, could overlook other medical evaluations (such as the pap test), considered erroneously not so necessary for their peculiar health condition. Nonetheless, also family physicians might undervalue the screening exams recommended for general population for their highly “medicalised” female patients. Thus, even though an increased incidence of cervical cancer cannot be unequivocally affirmed by our study, it should be remembered to pay attention also to this medical aspect in SSc women, in order to ensure the best medical care, even if not directly related to scleroderma.

## 6. Conclusions

In our SSc patients' series, we found a relatively high frequency of cancerous lesions of the cervix by means of pap test. A significant association between gynaecological malignancies and anti-Scl70 autoantibodies was also found. These preliminary findings need to be verified in larger controlled epidemiological studies. In general, periodic gynaecological screening for SSc patients is truly suggested.

## Figures and Tables

**Table 1:** Clinical features and pap test screening findings in 80 SSc patients.

	SSc series	Normal	Inflammation	Atypical cells/cancer	*p* values	
	(*n* = 80)	A (*n* = 55)	B (*n* = 20)	C (*n* = 5)	(A versus C)	(A + B versus C)
Age at onset	51.2 ± 12	51.2 ± 12	51.2 ± 12	65 (46–67)	0.052	0.06
SSc duration	7.9 ± 5.8	8 ± 5.8	8 ± 5.8	6 (1–13)	0.45	0.45
Skin subsets						
Limited	72 (90%)	50 (90.9%)	18 (90%)	4 (80%)	0.42	0.42
Diffuse	8 (10%)	5 (9%)	2 (10%)	1 (20%)	0.42	0.42
Serology						
Anti-Scl70	23 (28.7%)	17 (30.9%)	2 (10%)	4 (80%)	**0.046**	**0.022**
ACA	37 (46.2%)	28 (50.9%)	8 (40%)	1 (20%)	0.35	0.37
ANoA	14 (17.5%)	6 (10.9%)	8 (40%)	0	1	0.58
Digital ulcers	29 (36.2%)	21 (38.2%)	7 (35%)	1 (20%)	0.64	0.65
Interstitial lung disease	30 (37.5%)	22 (40%)	8 (40%)	0	0.15	0.15
FVC < 70%	4 (5%)	3 (5.4%)	0	1 (20%)	0.30	0.23
DLCO < 70%	46 (57.5%)	32 (58.2%)	12 (60%)	2 (40%)	0.65	0.65
PAH	0					
History of smoking	27 (33.7%)	16 (29.1%)	9 (45%)	2 (40%)	0.63	1
History of immunosuppressant	5 (6.2%)	4 (7.3%)	1 (5%)	0	1	1
Pap test						
Negative	55 (68.7%)					
Inflammatory alterations	20 (25%)					
Atypical cells/cancer	5 (6.2%)					

**Table 2:** Cervical or vulvar cancers in SSc patients: review of the literature.

First author, year [ref.]	Country	Type of study	patient-years^1^ (pts/follow-up)	Cervical cancer	Vulvar cancer
Duncan, 1979 [9]	Mayo Clinic, USA	Cohort study	(2,141/n.d.^2^)	10	1
Lee, 1983 [10]	Canada	Cohort study	(95/7.3 ± 6.6)	2	0
Kyndt, 1997 [11]	France	Cohort study	(123/median 4)	1	0
Hill, 2003 [12]	South Australia	Registry-based	(441/6.1 ± 2.8)	5	0
Chatterjee, 2005 [13]	Detroit area, USA	Registry-based	4,908	3 (2 *in situ*)	0
Derk, 2006 [14]	Pennsylvania, USA	Cohort study	3,775	4	1
Olesen, 2010 [15]	Denmark	Registry-based	16,003	9	0
Airo', 2011 [16]	Italy	Cohort study	4,041	1	1
Belloli, 2011 [17]	Italy	Cohort study	(112/n.d.^3^)	3^4^	0
Szekanecz, 2012 [18]	Hungary	Cohort study	(218/n.d.)	1	0
Moinzadeh, 2014 [19]^5^	United Kingdom	Cohort study	(2,177/median 12.8)	17 “gynaecological” cases	
Shah, 2015 [20]^6^	John Hopkins H, USA	Cross-sectional	(1,044/1990–2012)	3	1
*Present series*	*Italy*	*Cohort study*	*560*	*2 (1 in situ)*	*2 (1 in situ)*

(1) When the number of patient-years is not reported, number of patients (pts) and number of years of follow-up are indicated in parenthesis. (2) Authors reviewed histories of SSc patients seen between 1959 and 1975. (3) Authors indicated that the follow-up was approximately from 2004 to November 2009. (4) Uterine cancers. n.d.: not done. (5) This study considered the database of SSc patients followed at the Centre for Rheumatology and Connective Tissue Diseases of the Royal Free Hospital, London. The “gynaecological” cases of cancer were not better defined, and were not classified either before or after SSc onset. (6) This study evaluated the database of SSc patients observed at the John Hopkins Scleroderma Centre, Baltimore, in the period from 1990 to 2012. Other 5 cases of cancer of the endometrium were cited.

All SSc series studied included male patients, except our cohort.

In our study, 2 cases of cervical cancer and 2 cases of vulvar cancers were found, as reported; the further case who presented an endocervical polyp with immature squamous metaplasia is not indicated.
